# The contribution of sleep to anorexia nervosa severity

**DOI:** 10.1007/s40519-023-01531-w

**Published:** 2023-02-08

**Authors:** Tomoyuki Kawada

**Affiliations:** Department of Hygiene and Public Health, Nippon Medical School, 1-1-5 Sendagi, Bunkyo-Ku, Tokyo, 113-8602 Japan

I read with great interest the study by Malcolm et al. [1]. The authors examined the effect of sleep quality on subsequent anorexia nervosa (AN) symptom severity. Patients with AN have significantly poorer sleep, including sleep disturbances, sleep medications, and daytime dysfunction. Sleep quality and negative emotions, including depression, anxiety, and stress, significantly predicted AN symptom severity, independent of sex and body mass index. The authors cited a study by Bat-Pitault et al. that reported the association between sleep and AN subtypes, considering quality of life (QoL) and emotional status [2]. Moreover, I have some comments.

Bat-Pitault et al. compared sleep characteristics, sleepiness, and chronotype of patients with AN restrictive (ANR) and AN binge eating/purging (ANB/P) [2]. Patients with ANBP showed significantly greater sleep disturbances and sleepiness and high preference for an eveningness chronotype compared to that of patients with ANR. Additionally, the QoL was lower and emotionality was more negative in poor sleepers than that in good sleepers. Subjective sleep disturbance is a useful indicator for predicting AN severity, especially in patients with ANB/P. I speculate that poor sleep and QoL in patients with AN may lead to poor physical and mental health. Further studies are needed to specify the causal relationship between sleep, AN, and QoL.

Among several lifestyle factors, the problematic use of physical activity (PPA) is closely associated with subsequent risk in patients with poor sleep and their QoL [3]. Carpine et al. examined the PPA status in patients with AN [4], and PPA was significantly associated with eating disorders, body image concerns, anxiety, depression, lower psychological well-being, QoL, and impaired sleep. Although early-onset AN presented a lower PPA prevalence than that of standard-onset AN, clinical symptoms were more severe in early-onset AN. The authors recommended that PPA detection in patients with early-onset AN may be important to reduce the risk of several mental and physical symptoms. Correspondingly, physical and mental disorders are combined in patients with AN and commitment of eating habits and body image cannot be easily removed from their consciousness. Modifying PPA in their daily life would gradually release patients from eating disorder.
